# Injuries to the Flexor Metatarsi

**Published:** 1899-04

**Authors:** R. C. Moore

**Affiliations:** Kansas City, Mo.


### INJURIES TO THE FLEXOR METATARSI.

By R. C. Moore, D.V.S.,
KANSAS CITY, MO.

This muscle being the sole flexor of the metatarsal region as well as check-ligament, lesions are of particular importance. Their occurrence is far from being rare in the horse, and sometimes is noticed in bovines.

In herbivora this muscle is composed of two quite distinct parts attached to each other by tendinous intersections. The one part is muscular with tendinous insertions, the other a tendinous structure that might well be considered a check-ligament. The tendinous portion arises with the extensor pedis from the distal end of the femur, from a depression just above the articular margin, between the external condyle and trochlea, passes down through the superior tibial groove, clothed by a reflection of one of the synovial membranes of the stifle-joint, passes down over the external and anterior face of the tibia, where it connects and exchanges numerous fibres with the muscular portion and extensor pedis; gaining the anterior face of the astragalus it forms a ring for the passage of the tendon of the muscular part, and bifurcates, sending one tendon externally to the cuboid and one downward to the head of the large metatarsal. This portion forms a ligamentous connection between the femur and tarsus and metatarsus, and acts as a stay to prevent undue extension of the metatarsus on the tarsus and of the latter on the tibia.

Some authors ascribe as a part of its action to flexion of the tarsus and metatarsus during extension of the stifle; but they are, to my mind, in error, as extension of the stifle cannot increase the distance between its points of origin and insertion, but it is probable that it may possess this action to a limited degree during flexion of the stifle. The fleshy portion or the real muscle arises from the supero-external part of the tibia just below the groove, becomes tendinous at the distal end, passes through the ring formed by the tendinous part, and bifurcates, sending a large, strong tendon

[1] Read before the Missouri Valley Veterinary Association, February 27, 1899.

to the head of the large metatarsal and a small one internally to the cuneïform parvum.

These structures are subject to injuries extending all the way from a slight strain of the fibres of some part of their structure to complete rupture, or they may be severed by cutting instruments or fence-wire, or the contractile power of the muscular fibres destroyed by bruises.

*Symptoms.* The symptoms are so marked that once seen they will never be forgotten. If the animal be standing on the affected limb when you observe him, you will not detect any unsoundness, provided no external lesions exist, but the moment his weight is shifted from the foot it is drawn backward, the tarsus being extended on the tibia by contraction of gastrocnemi, which is the opposing force to the flexor metatarsi, and as a result the tendons of these muscles above their attachment to the summit of the os calcis is greatly relaxed, so much so that the skin of the region is corrugated or thrown into folds, and should you first see him in this position you could not help thinking the distal end of the tibia had sustained an oblique fracture, causing a shortening of the bone, but when you inspect it in its normal position, bearing its share of weight without deformity, the thought of fracture will be dismissed.

The next thought that will most likely be directed to the tendo-Achilles, but if we consider that this is the support of the posterior part of the hock, and any inability on its part must allow the hock to drop down with tarsal flexion the instant the weight is placed upon it, and no such symptoms are present, but on the contrary, the hock supports the weight with its usual firmness, we must likewise banish from our minds the possibility of injury to this structure or to the muscles operating through it. After cancelling these possibilities we look deeper into our case, and readily perceive the inability to flex the metatarsus and tarsus on the tibia, and knowing that but one structure performs this function we have proved beyond a doubt that the injury is to the flexor metatarsi. Percivall's *Hippopathology*, vol. iv., part ii., page 337, quotes from Solleysel a beautiful description of this accident, but he unfortunately ascribes it to the tendo-Achilles, and calls it the master sinew; but his description is so perfect I cannot resist the temptation to copy it: "This," says he, "is the biggest and most visible sinew in a horse's body, which by reason of a strain occasioned by hard riding, evil shoeing, going down a steep place, a slip or fall, or too heavy burden, may be relaxed, and sometimes disturbed with so much violence that it becomes movable like an unbent bow-string. When a horse walks the leg seems to hang at the hough because its motion is not regulated by the master sinew; and you would even sometimes imagine that the bone was broken. When a horse stands with his foot fixed on the ground, the hough being extended in its natural posture, there is so little appearance of any grief in the leg, that it seems perfectly sound; but if you handle the master sinew, you will find it more movable than that of the other leg; and if you make the horse move his hinder parts, you will immediately perceive the sinew to be as loose and infirm as if it were broken."

The causes are varied, but anything that will cause extreme extension of the tarsus and metatarsus is liable to injure the muscle or its tendons, more particularly the tendinous portion. Falling forward, dragging the hind legs behind him, the hind foot becoming engaged or fast and the horse straining violently to free it. Bruises from kicks, being run into by the wheel of a vehicle, sharp-cutting instruments, barbed-wire fences, etc. I have seen two cases where the entire structure was cut off near the distal end of the tibia on wire fences, and two cases where the muscles were bruised respectively by being run into and falling. The two latter recovered, but it was thought best to destroy both those cut on the wire.

*Prognosis* will depend very largely on the extent of the injury. Where complete rupture of the entire structure has occurred resolution can scarcely be expected, and the same will be true if they are torn loose from the bone, although in the worst cases if the foot is kept well forward recovery may take place.

*Treatment.* Perfect rest is essential, with slings for the horse, if he will bear them. The leg should be kept well forward by a cord secured to the fetlock and around the neck, hot fomentations to allay the inflammation, and stimulating liniments will be sufficient. Rest should be prolonged until all signs of the lesions have disappeared. If an external wound, antiseptics and astringents are required.

